# Hybrid Molecular Lateral Flow Assay Targeting the lipL32 Gene for Rapid and Highly Sensitive Detection of Pathogenic *Leptospira*

**DOI:** 10.3390/tropicalmed11100288

**Published:** 2026-10-12

**Authors:** Syahira Aulia Difa, Widoretno, Farida Dwi Handayani, Masafumi Yohda, Raissa Zahrantiara, Citra Maharani Sukma, Yudha Gusti Wibowo, Muhamad Sahlan

**Affiliations:** 1 Department of Chemical Engineering, Faculty of Engineering, University of Indonesia, Depok 16424, Indonesia; syahira.difa@ui.edu; 2 Center of Biomedical Research, Research Organizaion for Health, National Research and Innovation Agency (BRIN), Cibinong Science Center, Bogor 16911, Indonesiafari018@brin.go.id (F.D.H.); 3 Department of Biotechnology and Life Science, Tokyo University of Agriculture and Technology, Koganei 148-4588, Japan; yohda@cc.tuat.ac.jp; 4 Research Centre of Biomedical Science, Faculty of Medicine, University of Malaya, Lembah Pantai, Kuala Lumpur 50603, Malaysia; raissazahrantiara@gmail.com; 5 Department of Biology, Faculty of Natural Science and Mathematics, Gadjah Mada University, Sleman 55281, Indonesia; 6 Center for Green and Sustainable Materials, Institut Teknologi Sumatera, Way Hui, Lampung Selatan 35365, Indonesia; 7 Research Centre for Biomedical Engineering, Universitas Indonesia, Depok 16424, Indonesia; 8 Department of Interdisciplinary Engineering, Universitas Indonesia, Depok 16424, Indonesia

**Keywords:** hybrid detection, lateral flow assay, *Leptospira*, LipL32, molecular diagnostics

## Abstract

Leptospirosis remains a major neglected zoonotic disease in tropical and subtropical regions, where delayed diagnosis contributes to severe clinical outcomes and continued underrecognition. Although real-time PCR provides high diagnostic accuracy, its dependence on specialized instrumentation limits its use in resource-constrained endemic settings. This study developed and clinically evaluated a lipL32-targeted hybrid molecular lateral flow assay (Hybrid LFA) for visual detection of pathogenic *Leptospira* DNA in urine, plasma, and whole blood specimens. Thirty-five clinical samples, comprising 17 urine, 14 plasma, and 4 whole blood specimens, were processed using automated magnetic bead-based DNA extraction, followed by PCR amplification and lateral flow-based amplicon detection. Assay performance was compared with culture, real-time PCR, and conventional PCR. Real-time PCR showed complete agreement with culture, correctly identifying all 23 culture-positive and 12 culture-negative specimens. The Hybrid LFA detected 18 of 23 positive specimens, yielding an overall sensitivity of 78.26%, specificity of 100%, and diagnostic accuracy of 85.71%. Matrix-specific analysis showed the highest sensitivity in urine specimens (84.62%), followed by whole blood (75.00%) and plasma (57.14%), indicating that specimen type substantially influenced assay performance. In contrast, conventional PCR detected only 4 of 23 positive specimens, corresponding to a sensitivity of 17.39%. Analytical sensitivity testing demonstrated that the Hybrid LFA achieved a limit of detection of 1.13 × 10^−7^ ng/µL, approximately three orders of magnitude lower than agarose gel electrophoresis. These findings indicate that the lipL32-targeted Hybrid LFA is a highly specific PCR-coupled visual detection platform with promising potential for rapid leptospirosis screening, particularly using urine specimens. Larger prospective validation studies are required to confirm its clinical applicability in endemic and resource-limited settings.

## 1. Introduction

Leptospirosis is a globally distributed zoonotic bacterial disease and remains a major public health concern, particularly in tropical and subtropical regions where flooding, inadequate sanitation, poor infrastructure, and limited access to diagnostic and healthcare services contribute to disease transmission and burden [1,2,3]. Global estimates indicate that leptospirosis causes approximately 1.03 million cases and 58,900 deaths annually [4], corresponding to approximately 2.90 million disability-adjusted life years (DALYs) lost each year [5]. The clinical manifestations of leptospirosis are highly variable, ranging from asymptomatic or mild influenza-like illness to severe disease characterized by jaundice, acute kidney injury, pulmonary involvement, and multi-organ dysfunction [2,3]. During the early phase of infection, leptospirosis commonly presents as an acute undifferentiated febrile illness, with nonspecific manifestations that substantially overlap with those of dengue, malaria, typhoid fever, influenza, and other infectious diseases, thereby complicating clinical diagnosis, particularly in endemic regions [6,7]. Delayed recognition may postpone appropriate antimicrobial and supportive therapy and increase the likelihood that patients are diagnosed only after complications or organ dysfunction have developed [8]. These diagnostic challenges are particularly important in resource-limited endemic settings, where access to timely and accurate laboratory testing remains restricted.

Conventional laboratory diagnosis of leptospirosis relies primarily on culture and the microscopic agglutination test (MAT), although both approaches have important limitations for routine and early clinical diagnosis. Isolation of *Leptospira* by culture provides direct evidence of infection but is technically demanding, slow, and has low diagnostic sensitivity, substantially limiting its usefulness for timely clinical decision-making [2,9]. MAT remains the serological reference standard; however, it is labor-intensive, requires maintenance of a representative panel of live *Leptospira* cultures, skilled personnel, specialized laboratory facilities, and considerable expertise for result interpretation [9,10]. Moreover, because MAT detects the host antibody response rather than the pathogen itself, its sensitivity is particularly limited during the early phase of infection, when circulating antibody levels may remain below detectable thresholds. Consequently, confirmation frequently relies on paired acute- and convalescent-phase serum samples demonstrating seroconversion or a significant rise in antibody titer, which delays definitive diagnosis and limits the utility of MAT for early clinical decision-making [10,11].

Rapid diagnostic tests based on immunochromatographic and lateral flow technologies have been developed to provide simple, rapid, and relatively affordable point-of-care approaches for leptospirosis diagnosis. However, substantial variability in diagnostic performance has been reported across different assays and study populations. Clemente et al. [12] reported sensitivities ranging from 15.8% to 100% and specificities from 37.3% to 100% across immunochromatographic assays evaluated for acute human leptospirosis. Similarly, Maze et al. [13] reported pooled sensitivity and specificity estimates of 79% (95% CI: 70–86%) and 92% (95% CI: 85–96%), respectively, for whole-cell lateral flow assays, while emphasizing limitations related to study quality and the use of imperfect reference standards. More recently, Nualnoi et al. [14] reported pooled sensitivity and specificity of 68% (95% CI: 57–78%) and 93% (95% CI: 90–95%), respectively, across lateral flow immunoassays, with substantial between-study heterogeneity. Collectively, these findings indicate that currently available serological rapid tests exhibit variable diagnostic sensitivity and may therefore have limited reliability when used as stand-alone tests, particularly when early diagnosis is required.

Recent advances in molecular diagnostics have enabled the development of nucleic acid-based point-of-care platforms that combine direct pathogen detection with rapid and highly sensitive analytical readouts. Among the molecular targets investigated for leptospirosis diagnosis, the *lipL32* gene has been widely used because of its high sequence conservation among pathogenic *Leptospira* and its absence from saprophytic strains. *lipL32* encodes LipL32, a highly abundant outer membrane lipoprotein that is expressed during mammalian infection and has been implicated in interactions with host components, supporting its utility as a molecular diagnostic target [15,16]. Several studies have further demonstrated the feasibility of integrating nucleic acid amplification with CRISPR-based detection and lateral flow readouts. For example, Jirawannaporn et al. [17] developed an RPA-CRISPR/Cas12a platform targeting lipL32 with a limit of detection of 100 cells/mL and subsequently demonstrated lateral flow detection of amplified targets.

Despite these advances, clinically validated molecular point-of-care assays for leptospirosis remain limited, and their diagnostic performance across different clinical specimen types, stages of infection, and epidemiological settings remains incompletely characterized. Although molecular methods can directly detect *Leptospiral* DNA during the early leptospiremic phase, their performance is influenced by the timing of specimen collection, specimen type, pathogen load, and assay design [8,18]. In contrast, many currently available rapid diagnostic tests rely on the detection of host antibodies, which may be present at low or undetectable levels during the early phase of infection and therefore may limit diagnostic sensitivity before seroconversion [2,14]. Consequently, there remains a need for rapid, simple, and reliable molecular diagnostic platforms capable of directly detecting pathogenic *Leptospira* in clinically relevant specimens and that are suitable for implementation in resource-limited settings.

In this study, we developed and evaluated a hybrid molecular lateral flow assay targeting the *lipL32* gene for the rapid detection of pathogenic *Leptospira* in clinical specimens. The diagnostic performance of the developed assay was evaluated using clinical samples and compared with real-time PCR as the molecular reference method. By combining nucleic acid-based detection with a visually interpretable lateral flow readout, this approach was designed as a potential alternative molecular point-of-care platform for the early detection of leptospirosis, particularly in endemic and resource-limited settings.

## 2. Material and Method

### 2.1. Clinical Samples and Bacterial Isolate Material

A total of 35 de-identified clinical specimens were retrospectively obtained from collaborating institutions in Central Java, Indonesia, and included urine, plasma, and whole-blood samples. The specimens represented both positive and negative cases according to the reference diagnostic classification used in this study and were selected to evaluate the performance of the Hybrid LFA across different clinical matrices. Because the specimens were provided in a de-identified form, detailed patient demographic and clinical information, including disease duration, disease severity, and the timing of specimen collection, was not available for analysis.

Prior to molecular analysis, all clinical specimens underwent an initial preparation step to reduce particulate material and improve sample homogeneity. Briefly, samples were centrifuged at 8000 rpm for 10 min under sterile conditions, after which the supernatant and pellet fractions were processed according to the requirements of the subsequent analytical procedures. All 35 specimens were subjected to DNA extraction and subsequently analyzed using conventional PCR, real-time PCR, and Hybrid LFA.

Established bacterial isolates were included as positive controls throughout the experimental procedures to verify assay performance and support assessment of analytical specificity. Bacterial culture was used as the reference method for evaluating diagnostic performance because it provides direct evidence of viable *Leptospira* organisms. Although culture is constrained by prolonged incubation requirements and limited sensitivity, it remains an important microbiological confirmatory method for leptospirosis. Accordingly, the sensitivity, specificity, and diagnostic accuracy of the Hybrid LFA were calculated relative to culture results, while conventional PCR and real-time PCR were included as comparator molecular methods.

### 2.2. DNA Extraction Material

Genomic DNA was extracted using the HAS GENAU Nucleic Acid Extraction Kit (HAS GENAU, Central Jakarta, Indonesia) with the Skyfire automated nucleic acid extraction system (Skyfire, Hangzhou, China), following the manufacturer’s instructions. The extraction procedure was based on magnetic bead separation technology. Briefly, 200 µL of each sample was transferred into a sterile microcentrifuge tube and mixed with 20 µL of phosphate-buffered saline (PBS) prior to automated extraction. Purified DNA was eluted in a final volume of approximately 50 µL and subsequently divided into two 15 µL aliquots for downstream molecular analyses, while the remaining 20 µL was retained for repeat testing or storage.

### 2.3. PCR Amplification and Hybrid Lateral Flow Assay Detection

PCR amplification was performed to detect the pathogenic *Leptospira* lipL32 gene using the previously reported primer pair LipL32-F (5′-ATC TCC GTT GCA CTC TTT GC-3′) and LipL32-R (5′-ACC ATC ATC ATC ATC GTC CA-3′). This primer pair amplifies a 474-bp fragment of the lipL32 gene and was adopted from previously published studies rather than newly designed in the present work.

The PCR mixture contained 10 µL of Platinum II Hot-Start Green PCR Master Mix (2×) (Thermo Fisher Scientific, Vilnius, Lithuania), 0.4 µL of forward primer (10 µM), 0.4 µL of reverse primer (10 µM), 4 µL of Platinum GC Enhancer, and 5.2 µL of ddH_2_O. PCR amplification was performed using a two-step thermal cycling protocol consisting of an initial denaturation at 94 °C for 2 min, followed by 40 cycles of denaturation at 94 °C for 15 s and annealing at 58 °C for 30 s. The amplified products were subsequently held at 4 °C until further analysis.

Following amplification, the 474-bp lipL32 PCR product underwent the labeling/hybridization step required for Hybrid LFA detection. No independent oligonucleotide probe was designed or added in this study; detection relied on labeled amplification products generated during the PCR-based workflow. The treated amplification product was subsequently analyzed using the Milenia GenLine HybriDetect 1 system (Milenia Biotec GmbH, Giessen, Germany), consisting of HybriDetect dipsticks and the corresponding assay buffer. The dipstick was immersed in the prepared reaction mixture and incubated at ambient temperature, and the results were visually interpreted within 5–15 min.

A positive result was defined by the simultaneous appearance of both the test and control lines, whereas the appearance of the control line alone was interpreted as negative. The absence of the control line was considered an invalid test. Representative amplification products were additionally evaluated by agarose gel electrophoresis to confirm amplification of the expected 474-bp lipL32 fragment and to provide a reference for comparison with the Hybrid LFA readout.

Analytical sensitivity was evaluated using a 10-fold serial dilution series of target DNA comprising eight concentration levels: 1.13, 1.13 × 10^−1^, 1.13 × 10^−2^, 1.13 × 10^−3^, 1.13 × 10^−4^, 1.13 × 10^−5^, 1.13 × 10^−6^, and 1.13 × 10^−7^ ng/µL. Each dilution was subjected to the same PCR amplification procedure and subsequently assessed using agarose gel electrophoresis and Hybrid LFA detection. For agarose gel electrophoresis, the limit of detection (LOD) was defined as the lowest tested DNA concentration yielding a visually detectable band corresponding to the expected *lipL32* amplicon. For the Hybrid LFA, the LOD was defined as the lowest tested concentration producing a visible test line in the presence of a valid control line. Based on these criteria, the LODs were 1.13 × 10^−4^ ng/µL for agarose gel electrophoresis and 1.13 × 10^−7^ ng/µL for the Hybrid LFA.

### 2.4. Statistical Analysis

Diagnostic performance of the Hybrid LFA was evaluated against the microbiological reference classification using a 2 × 2 contingency table. True-positive (TP), true-negative (TN), false-positive (FP), and false-negative (FN) results were used to calculate sensitivity, specificity, positive predictive value (precision), and overall diagnostic accuracy, as follows:

$$sensitivity=\frac{TP}{\left(TP+FN\right)}\times 100\%$$

$$Specificity=\frac{TN}{\left(TN+FP\right)}\times 100\%$$

$$Precision=\frac{TP}{\left(TP+FP\right)}\times 100\%$$

$$Accuraty=\frac{\left(TP+TN\right)}{\left(TP+TN+FN\right)}\times 100\%$$

Agreement between the Hybrid LFA and the reference classification beyond chance was assessed using Cohen’s kappa coefficient (κ):

$$K=\frac{\left(P_{o}-P_{e}\right)}{\left(1-P_{e}\right)}$$

where *P_o_* represents the observed proportion of agreement and *P_e_* represents the expected proportion of agreement by chance based on the marginal totals of the contingency table. All calculations were performed using Microsoft Excel for Microsoft 365 (Microsoft Corporation, Redmond, WA, USA).

## 3. Result and Discussion

The development of a robust molecular diagnostic platform requires the integration of efficient sample processing and highly sensitive detection methods. In this study, the performance of a Hybrid Lateral Flow Assay was evaluated using diverse clinical matrices to establish its potential for rapid disease identification. The following sections detail the analytical outcomes of the genomic DNA extraction process, the optimization of the molecular parameters for real-time sensitivity, and the subsequent visual characterization using the LFA platform. These findings provide critical insights into the relationship between specimen quality and diagnostic performance in real-world clinical applications.

### 3.1. Yield and Purity Analysis of DNA Isolated from Clinical Specimens

The quality and integrity of extracted nucleic acids are critical preanalytical factors that can influence the analytical sensitivity, reproducibility, and reliability of molecular diagnostic assays. According to the updated Minimum Information for Publication of Quantitative Real-Time PCR Experiments (MIQE 2.0) guidelines, appropriate nucleic acid quality control should include assessment of concentration, integrity, and the potential presence of PCR inhibitors before amplification, as variation introduced during sample processing and nucleic acid extraction can substantially affect downstream qPCR performance [19]. To demonstrate the quality of extracted nucleic acids, representative DNA samples from selected urine and plasma specimens were characterized using microvolume UV–Vis spectrophotometry. Although DNA extraction was performed for all 35 clinical specimens, only selected samples are presented in Table 1 to illustrate variations in DNA concentration and purity among different clinical matrices.

As shown in Table 1, total DNA concentrations varied substantially among the clinical specimens, ranging from 7.1 ng/μL in plasma sample P81 to 82.9 ng/μL in urine sample U41. Such variation is expected among clinical matrices because specimen cellularity, host-derived nucleic acid content, matrix composition, extraction efficiency, and pathogen abundance can differ considerably between individuals and specimen types. Importantly, total DNA concentration does not directly represent the *Leptospira* burden within a specimen. Previous studies have demonstrated that the diagnostic yield of molecular testing for leptospirosis is strongly influenced by specimen type and the timing of sample collection relative to disease onset [18,20]. During the early leptospiremic phase, leptospires and their DNA are predominantly detectable in blood-derived specimens, whereas urinary shedding becomes increasingly relevant later in the course of infection, reflecting the transition toward the leptospiruric phase [21]. Consequently, the concentration of pathogen-derived DNA available for molecular detection may vary substantially among specimens and across different stages of infection. This variability, together with the frequently nonspecific clinical presentation and recognized underdiagnosis of leptospirosis, emphasizes the importance of sensitive molecular workflows capable of detecting low-abundance pathogenic *Leptospira* DNA in heterogeneous clinical specimens [2,11,22].

Despite the broad range of DNA concentrations observed, measurable total DNA was successfully recovered from all clinical specimens, indicating that the magnetic bead-based extraction procedure provided sufficient nucleic acid for subsequent molecular analysis. However, because the measured DNA represents total nucleic acid recovered from each specimen, including predominantly host-derived DNA, these concentrations should not be interpreted as a direct measure of *Leptospira* DNA recovery or bacterial burden. Efficient nucleic acid extraction nevertheless represents a critical pre-analytical determinant of molecular diagnostic sensitivity, particularly in leptospirosis, where the concentration of pathogenic *Leptospira* in clinical specimens may approach the detection limit of molecular assays [22,23]. Bourhy et al. [23] demonstrated that specimen type, input volume, elution volume, and DNA extraction procedure substantially influence the recovery and subsequent PCR detection of leptospiral DNA, particularly at low bacterial concentrations. Similarly, Nhan et al. [22] identified low bacterial load as a major challenge in PCR-based diagnosis and showed that inadequate DNA recovery can contribute to reduced molecular detection sensitivity. More recently, Uduwawala et al. [24] demonstrated that rapid magnetic bead-based nucleic acid extraction could be successfully integrated with isothermal amplification for the detection of pathogenic *Leptospira*, further emphasizing the importance of efficient sample preparation in molecular point-of-care workflows. Therefore, the recovery of measurable nucleic acid from all specimens in the present study provides an appropriate basis for subsequent amplification and Hybrid Lateral Flow Assay (Hybrid LFA) analysis, while pathogen-specific amplification remains necessary to confirm the presence and detectability of *Leptospira* DNA.

DNA purity was further assessed using the A260/A280 absorbance ratio, which is commonly used as an indicator of protein and other UV-absorbing contaminants in nucleic acid preparations. Most samples exhibited ratios within or close to the commonly accepted range of approximately 1.8–2.0 for relatively pure DNA, suggesting low levels of protein contamination and general suitability of the extracted nucleic acids for downstream molecular analysis [25,26]. Plasma samples P52 and P81 exhibited A260/A280 ratios of 1.99 and 2.07, respectively, while urine sample U79 displayed a ratio of 2.06. These values are consistent with relatively low protein carryover, although A260/A280 ratios slightly above 2.0 should not be interpreted as definitive evidence of superior DNA purity, as spectrophotometric purity ratios can be influenced by physicochemical factors such as pH and ionic strength of the measurement solution [26]. Moreover, spectrophotometric purity ratios should be interpreted cautiously for samples with low DNA concentrations, because measurement reproducibility decreases as nucleic acid concentrations approach the lower analytical range of UV-based methods [27,28,29]. High-quality nucleic acid templates are important for reliable amplification efficiency and inter-assay reproducibility; however, A260/A280 ratios alone cannot exclude the presence of PCR inhibitors, and assay-specific amplification controls are required to evaluate inhibition directly [19].

Sample U41 exhibited the highest total DNA concentration (82.9 ng/μL) but the lowest A260/A280 ratio (0.81), whereas sample U52 showed a moderately reduced ratio of 1.52. These values suggest the presence of residual protein or other co-extracted UV-absorbing contaminants. Urine is a complex clinical matrix that may contain PCR-inhibitory substances, including urea and other endogenous compounds capable of interfering with DNA polymerase activity [30]. Nevertheless, successful amplification from the urine specimens in the present study indicates that any residual inhibitors were not sufficient to completely prevent target amplification under the assay conditions used. However, because no dedicated internal inhibition control was used to quantify matrix-associated inhibition, complete removal or absence of PCR inhibitors cannot be assumed.

The relatively higher Hybrid LFA sensitivity observed in urine is also consistent with the potential diagnostic value of urinary detection of pathogenic *Leptospira*. Following renal dissemination, leptospires may be shed into the urine, and several molecular diagnostic studies have demonstrated that urine can provide useful or even complementary detection to blood-derived specimens, depending on the stage of infection and timing of sample collection [18,31]. Therefore, the higher sensitivity observed in urine in the present study may reflect differences in pathogen distribution or target availability between specimen types; however, this interpretation should remain cautious in the absence of quantitative bacterial-load measurements and standardized sampling times.

Importantly, suboptimal spectrophotometric purity ratios do not necessarily preclude successful molecular detection, because the inhibitory effect of co-extracted substances depends on their chemical identity and concentration, the sample preparation and nucleic acid extraction procedure, and the susceptibility of the specific amplification reaction to inhibition [30,32]. Studies of *Leptospira* molecular diagnostics have similarly shown that extraction efficiency and matrix-associated inhibition can influence analytical sensitivity and that internal controls are valuable for identifying inhibition-related false-negative results [33]. Thus, the successful amplification obtained from specimens with variable purity in the present study supports the practical compatibility of the extracted DNA with the downstream Hybrid LFA workflow, while not excluding the possibility of partial amplification inhibition.

Additional assessment using the A260/A230 absorbance ratio revealed values ranging from 0.10 to 0.77, substantially below the commonly expected range of approximately 2.0–2.2 for relatively pure nucleic acid preparations. Low A260/A230 ratios are generally associated with the presence of compounds absorbing near 230 nm, including residual chaotropic salts, EDTA, carbohydrates, phenolic compounds, and other reagents carried over during nucleic acid extraction [25]. The markedly reduced ratios observed in the present study therefore indicate that some co-extracted contaminants or extraction reagents remained in the purified nucleic acid preparations. However, spectrophotometric purity ratios alone do not directly predict the extent of PCR inhibition. Previous studies have shown that DNA extracts with suboptimal A260/A230 ratios may still support successful amplification, whereas the actual effect of contaminants depends on their chemical identity, concentration, and the susceptibility of the amplification system [25]. Accordingly, the successful downstream amplification observed in the present study indicates that residual contaminants did not completely prevent target detection under the assay conditions used; however, partial inhibition or reduced amplification efficiency cannot be excluded without dedicated inhibition controls or dilution-based assessment [19]. Thus, despite the low A260/A230 ratios, the extracted nucleic acids remained usable for subsequent amplification and Hybrid LFA analysis, while the potential influence of residual inhibitors should be considered when interpreting analytical sensitivity.

Urine samples exhibited greater variability in DNA concentration and purity than plasma specimens, which may reflect differences in matrix composition, host-derived nucleic acid content, and pathogen shedding. Following hematogenous dissemination, pathogenic *Leptospira* may localize to the kidneys and be released into the urine, making urine an important specimen for molecular diagnosis, particularly as the infection progresses [21]. Molecular testing of urine can therefore provide complementary diagnostic information to blood-based assays, because the relative detectability of *Leptospiral* DNA in different specimen types changes over the course of infection.

From a diagnostic perspective, the recovery of measurable total DNA from all clinical specimens, including samples with concentrations as low as 7.1 ng/μL, indicates that the extraction workflow consistently produced nucleic acid material suitable for subsequent molecular analysis. However, because these measurements represent total recovered DNA rather than pathogen-specific DNA, they should not be interpreted as direct evidence of efficient *Leptospira* recovery. Sample preparation remains a critical determinant of nucleic acid-based point-of-care performance because extraction efficiency, target recovery, nucleic acid integrity, and removal of amplification inhibitors directly influence downstream analytical sensitivity and reproducibility [34,35]. This consideration is particularly relevant to leptospirosis, where clinical specimens may contain low concentrations of pathogenic *Leptospira* DNA approaching the detection limits of molecular assays [23].

The variable diagnostic performance reported for conventional serological lateral flow assays further highlights the need for alternative diagnostic approaches with improved sensitivity and standardized analytical workflows [12,13,14]. A recent meta-analysis reported pooled sensitivity and specificity of 68% and 93%, respectively, for leptospirosis lateral flow immunoassays, with substantial between-study heterogeneity [14]. Therefore, the recovery of amplifiable nucleic acid from the specimens evaluated in the present study provides an appropriate basis for further assessment of amplification efficiency and visual detection using the developed Hybrid LFA platform. Optimization of nucleic acid recovery, inhibitor removal, and target amplification will remain important for maximizing the analytical performance of this molecular assay, particularly for specimens containing low levels of pathogenic *Leptospira* DNA.

### 3.2. Analytical Sensitivity and Limit of Detection of the Hybrid LFA Platform

The analytical sensitivity of the developed Hybrid LFA platform was evaluated by determining its limit of detection (LOD) and comparing its detection capability with conventional agarose gel electrophoresis. LOD represents the lowest target concentration that can be reliably distinguished and detected under defined assay conditions and is therefore a fundamental parameter for evaluating the analytical performance of molecular diagnostic assays [19,36]. Establishing the LOD is particularly important for leptospirosis diagnosis because pathogenic *Leptospira* may occur at relatively low concentrations in clinical specimens, especially during the early phase of infection, requiring highly sensitive molecular detection methods [23]. Accordingly, comparison of the Hybrid LFA with agarose gel electrophoresis provides an indication of whether the lateral flow readout can improve visualization of low-concentration amplification products and potentially extend the detectable range of the molecular assay.

The analytical performance of the present Hybrid LFA should also be considered in the context of previously reported molecular lateral flow platforms for *Leptospira* detection [37,38]. Earlier approaches have combined nucleic acid amplification with lateral flow readout using different molecular targets and amplification strategies, including PCR- and isothermal amplification-based systems [37,38]. These studies collectively demonstrate the potential of molecular LFA platforms to provide rapid and visually interpretable detection while reducing dependence on conventional electrophoretic or fluorescence-based readout systems. However, direct comparison of analytical sensitivity among these platforms requires caution because reported detection limits are influenced by differences in target genes, amplification chemistry, template type, DNA quantification methods, and the units used to define analytical sensitivity [19,39]. In the present study, targeting the pathogenic *Leptospira*-associated *lipL32* gene enabled detection down to 1 pg/µL, while maintaining specificity against the non-target bacterial species evaluated. Importantly, the proposed platform was further assessed using urine, plasma, and whole blood specimens, providing preliminary evidence of its applicability across clinically relevant sample matrices. Thus, rather than representing a direct replacement for previously reported molecular LFAs, the present Hybrid LFA extends the existing diagnostic framework by combining *lipL32*-targeted amplification, rapid visual detection, and preliminary multi-matrix clinical evaluation within a single diagnostic workflow. Table 2 shows the comparison study for pathogenic *Leptospira* detection.

**Figure 1:**
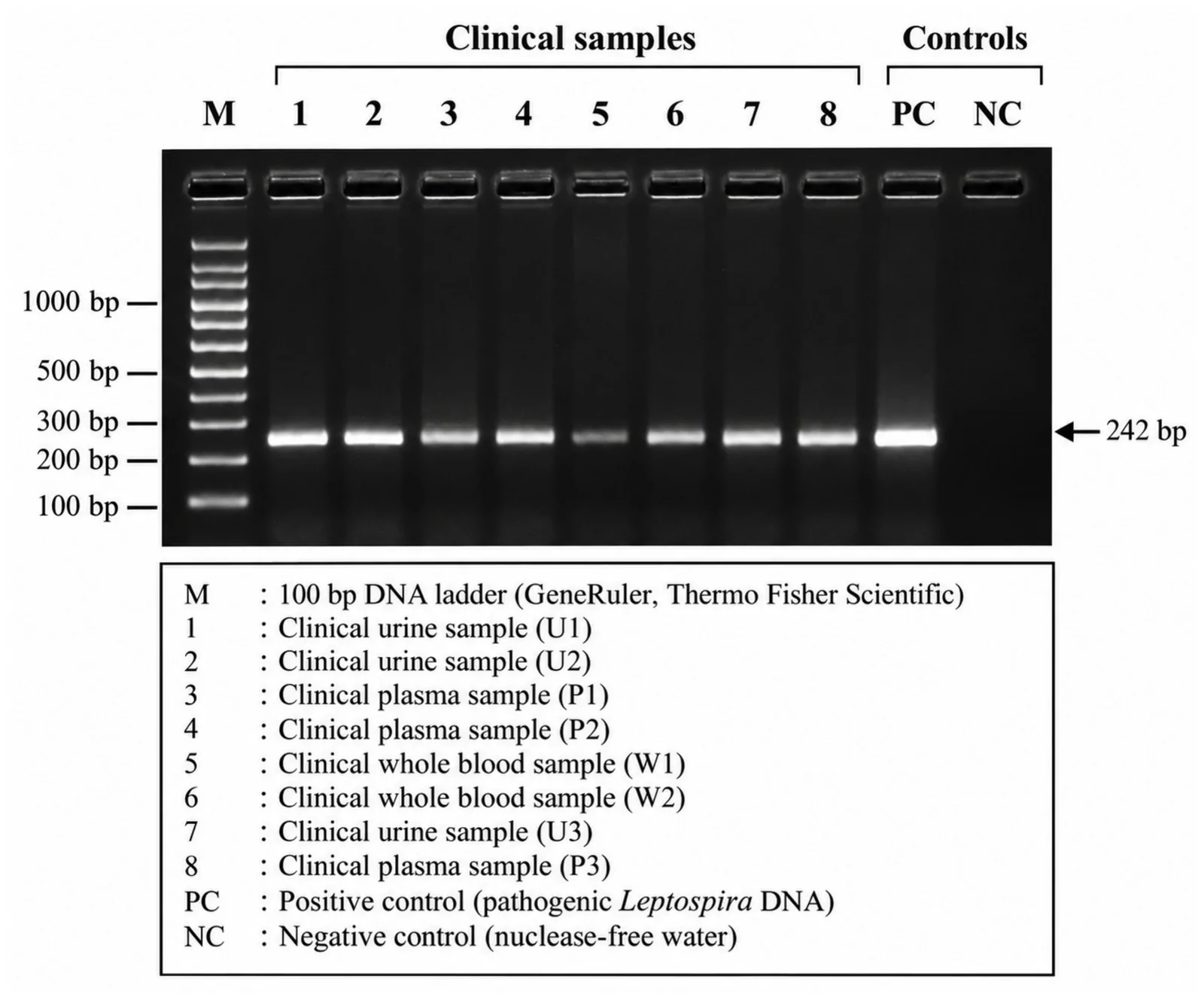
Representative agarose gel electrophoresis image showing the absence of target bands in negative samples (K − 57 to K − 59) and successful amplification of pathogenic *Leptospira* DNA in positive samples (K + 57 to K + 59).

Representative agarose gel electrophoresis results are shown in Figure 1. No detectable amplification products were observed in the negative controls (K − 57, K − 58, and K − 59), whereas distinct bands corresponding to the expected target amplicon were detected in all positive controls (K + 57, K + 58, and K + 59). These results support the specificity of the amplification reaction under the experimental conditions used and indicate that no detectable contamination or non-specific product of comparable size was present in the negative controls. However, the absence of visible bands in negative controls alone does not constitute comprehensive evidence of analytical specificity, which ideally requires evaluation against a broader panel of non-target organisms and sequence-confirmed target amplification. Previous molecular assays targeting the lipL32 gene have similarly demonstrated high analytical specificity for pathogenic *Leptospira* when evaluated against pathogenic, intermediate, saprophytic, and non-*Leptospira* microorganisms [33,42]. Thus, the clear discrimination between positive and negative controls observed in the present study provides initial evidence that the amplification step is suitable for subsequent integration with the Hybrid LFA platform.

The detection limit obtained using agarose gel electrophoresis was 1.13 × 10^−4^ ng/μL, whereas the Hybrid LFA detected the target at a concentration as low as 1.13 × 10^−7^ ng/μL. Under the experimental conditions used, this corresponds to a 1000-fold, or three orders of magnitude, lower detection threshold for the Hybrid LFA compared with conventional electrophoretic visualization. This finding indicates that the lateral flow readout provided substantially greater sensitivity for visualization of low-concentration amplification products. The improved detection capability of the Hybrid LFA may be attributed to efficient capture and localized accumulation of labeled amplification products at the test line, which generates a concentrated visual signal that can remain detectable at target concentrations below the visualization threshold of agarose gel electrophoresis. Similar improvements in amplicon detection have been reported for nucleic acid lateral flow platforms, where optimized capture chemistry and signal-generating labels enable lower detection limits than conventional gel-based visualization [40,41,42,43]. Nevertheless, because the present comparison primarily evaluates the sensitivity of two post-amplification readout methods, the observed 1000-fold difference should be interpreted as an improvement in amplicon detection sensitivity rather than an equivalent improvement in the intrinsic amplification efficiency of the PCR assay. The analytical sensitivity comparison between agarose gel electrophoresis and Hybrid LFA is summarized in Table 3.

The enhanced analytical sensitivity provided by the Hybrid LFA is potentially advantageous for leptospirosis diagnosis because pathogenic *Leptospira* DNA may be present at low abundance in clinical specimens, particularly in patients with low-level leptospiremia. Molecular assays with insufficient analytical sensitivity may consequently fail to detect specimens containing target concentrations near or below the assay detection threshold, potentially contributing to false-negative results. Accordingly, improving analytical sensitivity while retaining rapidity and operational simplicity represents an important objective in the development of molecular point-of-care diagnostics for leptospirosis [22,23]. Recent advances in biosensor technology have demonstrated that coupling nucleic acid amplification with lateral flow detection can substantially enhance the detectability of low-abundance targets while preserving the rapid and user-friendly characteristics of lateral flow platforms. Improvements in reporter systems, nanoparticle-based labels, capture-probe design, target preconcentration, and dedicated signal-enhancement strategies have all contributed to lower detection limits in advanced lateral flow assays [39,44]. These studies demonstrate the potential of amplification-assisted and CRISPR-integrated lateral flow platforms for highly sensitive molecular detection. However, reported detection limits across different platforms should be compared cautiously because they depend on target definition, nucleic acid preparation, amplification strategy, sample volume, and the units used to express analytical sensitivity.

### 3.3. Comparative Diagnostic Performance of Real-Time PCR, Hybrid LFA, and Conventional PCR

The diagnostic performance of the developed Hybrid LFA platform was evaluated using 35 clinical specimens comprising urine, plasma, and whole blood samples. Bacterial culture was used as the microbiological reference standard because it confirms viable *Leptospira* organisms and remains the conventional reference method in leptospirosis diagnostic studies. Real-time PCR and conventional PCR were included as comparator molecular assays to benchmark the performance of the developed Hybrid LFA against established nucleic acid amplification methods. The comparative results are summarized in Table 4.

Real-time PCR showed complete agreement with the culture results in the specimens evaluated. All 23 culture-positive samples were also positive by real-time PCR, whereas all 12 culture-negative samples were negative by real-time PCR, corresponding to a positive percent agreement of 100% (95% CI: 85.2–100%) and a negative percent agreement of 100% (95% CI: 73.5–100%). The overall agreement between the two methods was therefore 100% in this dataset. These findings support the suitability of real-time PCR as the molecular comparator method for evaluating the diagnostic performance of the Hybrid LFA. The strong performance observed is consistent with previous studies demonstrating high analytical sensitivity and specificity of real-time PCR assays targeting the lipL32 gene [33,42]. Stoddard et al. reported a 95% detection limit of approximately 20 genomic equivalents per reaction and selective detection of pathogenic *Leptospira*, whereas Ahmed et al. reported analytical detection down to approximately one genomic copy per reaction under optimized conditions, together with diagnostic sensitivity and specificity of 93.0% and 98.3%, respectively [33,42]. Real-time PCR is particularly useful during early infection because it enables direct detection of *Leptospiral* DNA before seroconversion and can therefore complement conventional serological methods.

Although isolation of viable *Leptospira* by culture provides direct microbiological evidence of infection, its diagnostic utility is limited by slow organism growth, prolonged turnaround time, and relatively low recovery rates. Culture should therefore be regarded as a highly specific but insensitive comparator rather than an infallible diagnostic gold standard. Consequently, the complete agreement observed in the present study demonstrates strong concordance between real-time PCR and culture within the evaluated sample set, while broader estimates of clinical sensitivity and specificity would require validation against a larger cohort using a composite or otherwise appropriate reference standard.

Although bacterial culture is less sensitive than molecular methods, it was intentionally used in the present study as a microbiological reference comparator because isolation of viable *Leptospira* provides direct and highly specific evidence of infection. However, culture-negative results should be interpreted cautiously because the method has inherently low sensitivity and may fail to recover organisms from specimens containing low bacterial burdens or collected after antimicrobial treatment. Therefore, the complete agreement observed between culture and real-time PCR in the present dataset supports the internal consistency of the reference classification, particularly among culture-positive specimens, but does not establish culture as an infallible diagnostic gold standard [45].

In contrast, conventional PCR detected only 4 of the 23 culture-positive specimens, corresponding to a positive percent agreement of 17.39% with culture (95% CI: 4.95–38.78%). Positive conventional PCR results were obtained from three whole-blood specimens (W2, W3, and W4) and one plasma specimen (P73), whereas none of the culture-positive urine specimens were detected. The substantially lower detection rate of conventional PCR is likely multifactorial and may reflect lower analytical sensitivity of the specific assay, differences in target and primer design, reliance on end-point amplicon visualization, low concentrations of *Leptospiral* DNA, and matrix-associated inhibition. Similar findings have been reported previously [30,46,47]. Podgoršek et al. demonstrated that *lipL32*-targeted real-time PCR detected substantially more positive clinical specimens than conventional PCR, whereas a systematic review by Yang et al. showed wide variability in conventional PCR sensitivity, particularly across different specimen types [46,47]. Low target abundance and inhibitory substances present in clinical matrices may further reduce amplification success, especially when target DNA concentrations approach the analytical detection limit [23,48]. Thus, the low conventional PCR detection rate observed in the present study is consistent with the recognized limitations of end-point molecular detection in specimens containing low-abundance pathogenic *Leptospira* DNA.

The Hybrid LFA platform successfully detected 18 out of 23 positive specimens, yielding an overall clinical sensitivity of 78.26%, which represented a substantial improvement over conventional PCR. Importantly, no false-positive results were observed among the 12 negative specimens, resulting in a specificity of 100%. The overall diagnostic accuracy reached 85.71%, demonstrating that the Hybrid LFA platform provided reliable detection while maintaining excellent specificity. The superior performance of Hybrid LFA was particularly evident in urine samples, where 11 out of 13 culture-positive specimens were correctly identified, corresponding to a matrix-specific sensitivity of 84.6%. The performance observed in this study demonstrates the potential of Hybrid LFA as a PCR-integrated detection approach for pathogenic *Leptospira*, providing visual interpretation of amplified DNA products without requiring specialized signal-reading instruments. Plasma samples also showed satisfactory diagnostic performance, whereas one false-negative result was observed among whole-blood specimens. The relatively higher sensitivity observed in urine may be related to renal localization and urinary shedding of pathogenic *Leptospira*, which can increase the availability of *Leptospiral* DNA in this specimen type during certain stages of infection. Recent clinical evidence supports the diagnostic value of urine PCR, with Le Turnier et al. [31] reporting an overall sensitivity of 83.6% for urine PCR compared with 70.1% for blood PCR, and similarly high urine-PCR sensitivity during the first week of illness. However, the relative diagnostic yield of urine and blood is influenced by the timing of specimen collection and assay characteristics [18]. Therefore, the higher sensitivity observed in urine in the present study should be interpreted as consistent with specimen-dependent differences in pathogen distribution rather than as definitive evidence of greater bacterial burden in urine.

Five culture-positive specimens (U42, P45, P46, P52, and W1) were not detected by the Hybrid LFA platform. These false-negative results were observed across different specimen matrices, including urine, plasma, and whole blood, suggesting that assay performance was influenced by multiple factors rather than a single matrix effect. One possible explanation is that the target *Leptospira* DNA concentration in these specimens was close to the analytical detection threshold of the visual lateral flow readout, resulting in insufficient signal generation despite a successful amplification workflow. In addition, biological matrices such as plasma and whole blood may contain residual components capable of affecting amplification efficiency or downstream detection. Importantly, all culture-negative specimens remained negative by Hybrid LFA, indicating that the reduced sensitivity was associated primarily with failure to detect low-level positive samples rather than non-specific signal generation. Future investigations incorporating quantitative bacterial load assessment, replicate Hybrid LFA testing, and optimization of signal amplification strategies may further clarify the factors contributing to false-negative outcomes.

The false-negative Hybrid LFA results observed in some PCR-positive specimens are unlikely to be solely attributed to inefficient DNA extraction, as the same extracted DNA was successfully amplified by conventional PCR. Instead, these discrepancies may reflect differences in the analytical detection mechanisms between the two methods. Conventional PCR coupled with agarose gel electrophoresis detects amplified DNA fragments through post-amplification staining, whereas Hybrid LFA requires sufficient quantities of labeled amplicons to produce a visible immunochromatographic signal. Therefore, samples containing low target abundance or amplification products near the detection threshold may generate detectable PCR bands but insufficient visual signals on the Hybrid LFA strip. Residual contaminants originating from biological specimens or introduced during nucleic acid extraction can interfere with downstream amplification and reduce molecular assay sensitivity, particularly when target concentrations approach the analytical detection limit. Clinical matrices may contain endogenous PCR inhibitors, including hemoglobin, immunoglobulins, anticoagulants, and urea, which can impair polymerase activity and contribute to false-negative reactions [30]. Future optimization of labeling efficiency, signal amplification chemistry, and analytical sensitivity evaluation using quantitative target measurements may further improve Hybrid LFA detection performance.

Although the sensitivity of the Hybrid LFA was lower than that of real-time PCR, its observed diagnostic performance was within the broad range reported for previously developed rapid tests for leptospirosis. Systematic reviews have demonstrated substantial heterogeneity in the performance of immunochromatographic and lateral flow assays, with reported sensitivities ranging from 15.8% to 100% and specificities from 37.3% to 100% [12]. More recently, Nualnoi et al. reported pooled sensitivity and specificity estimates of 68% (95% CI: 57–78%) and 93% (95% CI: 90–95%), respectively, for leptospirosis lateral flow immunoassays, while emphasizing considerable between-study heterogeneity [14]. These findings provide useful context for the performance of the present Hybrid LFA; however, direct comparison should be interpreted cautiously because most previously evaluated rapid tests are serological assays, whereas the present platform detects amplified pathogen-derived nucleic acid.

In the present study, no false-positive Hybrid LFA results were observed among the evaluated negative specimens, corresponding to an observed specificity of 100% within this sample set. However, given the limited number and diversity of negative specimens, this finding should not be interpreted as evidence of universally perfect specificity. Further improvements in molecular lateral flow performance may be achieved through optimization of nucleic acid recovery, amplification efficiency, capture-probe design, reporter chemistry, and signal-enhancement strategies, all of which have been identified as important determinants of analytical sensitivity in advanced lateral flow platforms [39,44].

The Hybrid LFA successfully detected 18 of the 23 culture-positive specimens, while all 12 culture-negative specimens were correctly classified as negative. Accordingly, the assay yielded a sensitivity of 78.26% (18/23), specificity of 100% (12/12), precision of 100% (18/18), and overall diagnostic accuracy of 85.71% (30/35). Cohen’s Kappa analysis yielded a κ value of 0.712, indicating substantial agreement between the Hybrid LFA and the microbiological reference classification beyond that expected by chance. These findings demonstrate that, although several false-negative results reduced sensitivity, the Hybrid LFA maintained strong overall concordance with the reference classification and produced no false-positive results within the evaluated sample set.

### 3.4. Matrix-Specific Diagnostic Performance of Hybrid LFA in Urine, Plasma, and Whole Blood Specimens

To evaluate the performance of the developed Hybrid LFA across different biological matrices, a matrix-specific analysis was performed using urine, plasma, and whole-blood specimens, and the comparative results are summarized in Table 5. Clinical specimen composition can substantially influence molecular diagnostic performance through differences in nucleic acid recovery, target abundance, and the presence of endogenous amplification inhibitors [18,34]. These matrix-dependent effects are particularly relevant to leptospirosis because the distribution and detectability of pathogenic *Leptospira* vary among blood-derived specimens and urine, while inhibitory effects may also differ between specimen types [18]. In molecular lateral flow systems, reliable analytical performance therefore depends not only on the lateral flow detection chemistry but also on efficient upstream sample preparation and amplification prior to visual readout [39].

Matrix-specific analysis demonstrated that urine specimens exhibited the highest Hybrid LFA sensitivity, with 11 of 13 culture-positive samples correctly identified (84.62%), followed by whole blood (3/4, 75.00%) and plasma (4/7, 57.14%). The false-negative results consisted of two urine samples, three plasma samples, and one whole blood sample. These differences suggest that specimen matrix characteristics and target availability may influence assay performance. In contrast, plasma samples yielded a lower sensitivity of 57.14%, whereas whole blood specimens exhibited an intermediate sensitivity of 75.0%. These findings indicate that specimen type plays an important role in determining the diagnostic performance of the platform.

The relatively higher performance observed in urine specimens may be associated with renal localization and urinary shedding of pathogenic *Leptospira*, which can increase the availability of *Leptospiral* DNA in this specimen type during certain stages of infection. Following hematogenous dissemination, leptospires may reach the kidneys and enter the renal tubular compartment, from which organisms or their DNA can subsequently be detected in urine [21]. Urine therefore provides a valuable complementary matrix for molecular diagnosis alongside blood-derived specimens. Clinical studies further support the diagnostic value of urine-based molecular testing. Woods et al. demonstrated that the diagnostic performance of *Leptospira* qPCR varies according to specimen type and showed that urine can provide complementary detection to blood-derived samples [18]. Notably, urine PCR also showed high sensitivity during the first week of illness, indicating that urinary molecular detection may be useful earlier than traditionally assumed. Nevertheless, the relative performance of urine and blood is influenced by the timing of specimen collection, assay design, and patient population. Thus, the higher Hybrid LFA sensitivity observed in urine in the present study is consistent with specimen-dependent differences in *Leptospiral* distribution and target availability, although confirmation using quantitative bacterial-load data and standardized sampling times would be required to establish the underlying mechanism.

Although urine is a chemically complex matrix containing urea, salts, metabolites, cellular material, and other endogenous components, these constituents did not prevent molecular detection in most urine specimens evaluated in the present study. Nevertheless, two false-negative results were observed. Such discrepancies may reflect variation in *Leptospiral* DNA abundance, urine pH, specimen handling, or residual matrix-associated inhibitors that affect nucleic acid amplification. Urea is a recognized PCR inhibitor, and previous work on *Leptospira* detection from urine has demonstrated that urine pH and sample-processing conditions can substantially influence amplification efficiency [30,49]. In particular, acidic urine and inadequate removal of cellular material or other inhibitory compounds have been associated with reduced PCR detection, whereas appropriate pretreatment can improve analytical sensitivity. Matrix effects are also recognized as an important consideration in lateral flow systems, for which sample pretreatment and buffer optimization can improve target recovery and signal reproducibility [50].

Plasma specimens showed the lowest Hybrid LFA sensitivity among the matrices evaluated in the present study. Blood-derived matrices can contain endogenous or treatment-related PCR inhibitors, including immunoglobulins, lactoferrin, and anticoagulants such as heparin, which may reduce amplification efficiency when incompletely removed during nucleic acid extraction [30]. Therefore, the lower detection rate observed in plasma may reflect a combination of target abundance, specimen timing, extraction efficiency, and residual inhibition rather than a single matrix-specific mechanism. Because the Hybrid LFA detects amplified nucleic acid following extraction and PCR, direct interference of native plasma constituents with lateral flow signal generation cannot be assumed without additional matrix-effect experiments. Whole-blood specimens demonstrated intermediate performance, with three of four positive samples detected by the Hybrid LFA. Whole blood is a challenging matrix for nucleic acid amplification because hemoglobin and immunoglobulin G can inhibit DNA polymerase activity and reduce amplification efficiency [51]. However, the small number of whole-blood specimens evaluated in the present study limits conclusions regarding matrix tolerance. The detection of three positive samples nevertheless indicates that the extraction and amplification workflow was compatible with most of the whole-blood specimens tested, although residual inhibition cannot be excluded.

No false-positive Hybrid LFA results were observed among the negative specimens evaluated across urine, plasma, and whole blood. This finding indicates that no detectable matrix-associated non-specific signals occurred within the tested sample set. However, the limited number and diversity of negative specimens do not permit definitive conclusions regarding analytical specificity or cross-reactivity. Comprehensive specificity assessment would require larger negative cohorts and testing against clinically relevant non-target microorganisms. Molecular lateral flow platforms rely on sequence-specific amplification and capture mechanisms that can provide high selectivity, but overall specificity ultimately depends on primer design, probe or capture-sequence specificity, amplification conditions, and appropriate control panels [39].

Although the matrix-specific analysis provides preliminary insights into the diagnostic performance of the Hybrid LFA across different clinical specimens, these findings should be interpreted with caution. In particular, the whole blood evaluation included only four clinical specimens, which limits the statistical robustness of the calculated sensitivity and precludes definitive conclusions regarding assay performance in this matrix. Similarly, the overall cohort comprised only 35 clinical specimens, reflecting the proof-of-concept nature of the present study rather than a comprehensive clinical validation. Future investigations should include substantially larger and more diverse patient cohorts from multiple endemic regions to enable rigorous statistical evaluation, assess inter-individual variability, and establish clinically reliable estimates of diagnostic accuracy across different stages of leptospirosis.

### 3.5. Study Limitation and Recommendations

The present study represents an initial evaluation of Hybrid LFA performance using clinical specimens. Although promising detection capability was demonstrated, comprehensive validation following diagnostic guidelines requires larger prospective cohorts with predefined inclusion criteria and diverse clinical populations. The occurrence of false-negative results indicates that Hybrid LFA sensitivity remains influenced by the amount of detectable labeled amplicon and the visual signal generation threshold. As antibiotic administration is common in leptospirosis patients, future studies should evaluate whether prior treatment affects Hybrid LFA performance due to rapid reduction of circulating bacterial DNA. Additional analytical specificity studies involving larger negative cohorts and potential interfering organisms are required.

In addition, the preliminary clinical evaluation was based on a relatively small number of specimens, which limits the statistical precision and generalizability of the observed diagnostic performance. Accordingly, the reported sensitivity and specificity should be interpreted as descriptive estimates within the evaluated sample set rather than definitive population-level measures of diagnostic accuracy. The limited sample size also precluded robust estimation of confidence intervals and clinically generalizable predictive values. Future prospective studies involving larger, appropriately powered cohorts and representative disease prevalence are therefore required to establish precise estimates of sensitivity, specificity, positive and negative predictive values, and their corresponding confidence intervals.

The clinical specimens evaluated in this study were obtained from available archived samples and were not derived from a prospectively designed cohort. Therefore, potential variations in disease stage, bacterial burden, and previous clinical management could not be fully controlled. Future studies involving larger prospective cohorts with predefined inclusion criteria and detailed clinical metadata are required to establish the diagnostic performance of Hybrid LFA under routine clinical conditions. Another limitation of this study is the limited number of negative clinical specimens included for specificity evaluation. Although no false-positive results were observed among the tested negative samples, additional studies involving larger numbers of negative specimens and samples containing potential interfering microorganisms or matrix components are required to fully characterize cross-reactivity and analytical specificity. Future validation studies should incorporate repeated independent interpretation of Hybrid LFA results by multiple observers to enable formal assessment of inter-observer and intra-observer agreement using attribute agreement analysis. Such evaluation would provide additional evidence regarding the repeatability, reproducibility, and robustness of qualitative positive/negative interpretation of the Hybrid LFA platform.

## 4. Conclusions

A hybrid molecular lateral flow assay targeting the *lipL32* gene was successfully developed as a rapid and highly sensitive platform for the detection of pathogenic *Leptospira*. The developed assay demonstrated a clinical sensitivity of 78.26%, specificity of 100%, and an overall diagnostic accuracy of 85.71%, with the highest sensitivity observed in urine specimens (84.62%). In addition, the Hybrid LFA achieved a detection limit of 1.13 × 10^−7^ ng/µL, representing an approximately three-order-of-magnitude improvement in analytical sensitivity compared with conventional agarose gel electrophoresis. These findings highlight the potential of the developed platform as a reliable molecular point-of-care diagnostic tool for early leptospirosis detection, particularly in endemic and resource-limited settings. Although the developed Hybrid LFA demonstrated promising diagnostic performance and excellent specificity, the present findings should be interpreted within the context of the study limitations, including the relatively small sample size and the limited number of whole blood specimens. Consequently, larger multicenter prospective studies involving diverse patient populations and balanced specimen types are required to further validate the clinical performance, reproducibility, and diagnostic utility of the proposed platform before routine clinical implementation.

## Figures and Tables

**Table 1:** Representative DNA concentration and purity profiles of selected clinical urine and plasma specimens after genomic DNA extraction.

No	Sample		DNA Concentration (ng/μL)	Purity Ratio (A260/A280)	Contamination from Non-Protein Compounds (A260/A230)
	Type	ID			
1	Urine	U42	11.7	1.71	0.13
2	Urine	U50	77.2	1.58	0.77
3	Plasma	P46	12.4	1.80	0.19
4	Urine	U85	8.5	1.57	0.28
5	Urine	U18	24.2	1.87	0.67
6	Urine	U48	11.0	1.87	0.32
7	Plasma	P85	8.4	1.76	0.11
8	Plasma	P79	11.1	1.86	0.12
9	Urine	U41	82.9	0.81	0.44
10	Urine	U52	14.3	1.52	0.34
11	Plasma	P42	8.9	1.73	0.13
12	Plasma	P52	8.2	1.99	0.10
13	Urine	U79	11.0	2.06	0.33
14	Plasma	P81	7.1	2.07	0.10
15	Urine	U45	25.8	1.60	0.42
16	Plasma	P73	14.6	1.71	0.17

**Table 2:** Comparison of molecular lateral-flow and related rapid molecular assays for pathogenic *Leptospira* detection.

Molecular Target	Amplification/Recognition Strategy	Detection/Readout	Reported Analytical Sensitivity/LOD	Clinical Evaluation	Key Characteristics	Study
Pathogenic *Leptospira* DNA targets	Multiplex LAMP (m-LAMP)	Gold nanoparticle lateral-flow dipstick	Reported high analytical sensitivity with visual endpoint detection	Evaluated for pathogenic *Leptospira* detection	Isothermal amplification with simultaneous target and internal-control detection on a lateral-flow dipstick	[37]
*lipL32*	Recombinase polymerase amplification (RPA)	RPA compatible with rapid molecular detection/lateral-flow endpoint configuration	Approximately a few genomic equivalents per reaction under optimized conditions	Evaluated using clinical samples	Rapid isothermal amplification at approximately 37–39 °C, reducing dependence on conventional thermocycling	[40]
*lipL32*	PCR + dFnCas9/CRISPR recognition	Quantitative lateral-flow immunoassay	1 fg/mL (0.001 pg/mL)	Primarily analytical validation using *Leptospira* DNA/reference material	Very high analytical sensitivity; CRISPR-mediated target recognition; requires dFnCas9/gRNA and quantitative strip-reader configuration	[38]
*lipL32*	RPA-NALFIA	Nucleic acid lateral-flow immunoassay	Rapid detection reported within approximately 1 h	Pathogenic *Leptospira* evaluation	Integrates isothermal amplification with lateral-flow detection and reduces dependence on sophisticated instrumentation	[41]
*lipL32*	PCR amplification + labeled molecular recognition	Hybrid LFA, visual lateral-flow readout	1 pg/µL	35 clinical specimens: urine, plasma, and whole blood	Combines molecular amplification with simple visual readout and preliminary multi-matrix clinical evaluation	Present study

**Table 3:** Analytical sensitivity and limit of detection comparison between agarose gel electrophoresis and hybrid LFA detection.

Detection Method	Limit of Detection (ng/μL)
Agarose Gel Electrophoresis	1.13 × 10^−4^
Hybrid Lateral Flow Assay	1.13 × 10^−7^
Sensitivity Improvement	±10^3^

**Table 4:** Comparative evaluation of hybrid LFA against real-time PCR and conventional PCR.

No	Sample		Culture	Conventional PCR	Real-Time	Hybrid LFA
	Type	ID				
1	Urine	U45	Positive	Negative	Positive	Positive
2	Plasma	P81	Positive	Negative	Positive	Positive
3	Plasma	P73	Positive	Positive	Positive	Positive
4	Urine	U79	Positive	Negative	Positive	Positive
5	Plasma	P42	Positive	Negative	Positive	Positive
6	Urine	U52	Positive	Negative	Positive	Positive
7	Urine	U41	Positive	Negative	Positive	Positive
8	Plasma	P79	Positive	Negative	Positive	Positive
9	Plasma	P85	Positive	Negative	Positive	Positive
10	Urine	U48	Positive	Negative	Positive	Positive
11	Urine	U18	Positive	Negative	Positive	Positive
12	Urine	U85	Positive	Negative	Positive	Positive
13	Urine	U50	Positive	Negative	Positive	Positive
14	Urine	U42	Positive	Negative	Positive	Negative
15	Plasma	P45	Positive	Negative	Positive	Negative
16	Plasma	P46	Positive	Negative	Positive	Negative
17	Plasma	P52	Positive	Negative	Positive	Positive
18	Urine	U11	Positive	Negative	Positive	Positive
19	Urine	U21	Positive	Negative	Positive	Positive
20	Whole Blood	W1	Positive	Negative	Positive	Negative
21	Whole Blood	W2	Positive	Positive	Positive	Positive
22	Whole Blood	W3	Positive	Positive	Positive	Positive
23	Whole Blood	W4	Positive	Positive	Positive	Positive
24	Plasma	P1	Negative	Negative	Negative	Negative
25	Plasma	P2	Negative	Negative	Negative	Negative
26	Plasma	P3	Negative	Negative	Negative	Negative
27	Plasma	P4	Negative	Negative	Negative	Negative
28	Plasma	P5	Negative	Negative	Negative	Negative
29	Plasma	P6	Negative	Negative	Negative	Negative
30	Urine	U1	Negative	Negative	Negative	Negative
31	Urine	U2	Negative	Negative	Negative	Negative
32	Urine	U3	Negative	Negative	Negative	Negative
33	Urine	U4	Negative	Negative	Negative	Negative
34	Urine	U5	Negative	Negative	Negative	Negative
35	Urine	U6	Negative	Negative	Negative	Negative

**Table 5:** Matrix-specific performance of the Hybrid LFA platform across urine, plasma, and whole blood clinical specimens.

Clinical Matrix	Total Samples	Culture Positive	Hybrid LFA Positive (TP)	False Negative (FN)	Sensitivity (%)
Urine	19	13	11	2	84.62
Plasma	12	7	4	3	57.14
Whole blood	4	4	3	1	75.00

## Data Availability

The original contributions presented in this study are included in the article. Further inquiries can be directed to the corresponding author.
